# A *Toxoplasma gondii* Ortholog of *Plasmodium* GAMA Contributes to Parasite Attachment and Cell Invasion

**DOI:** 10.1128/mSphere.00012-16

**Published:** 2016-02-10

**Authors:** My-Hang Huynh, Vern B. Carruthers

**Affiliations:** Department of Microbiology and Immunology, University of Michigan School of Medicine, Ann Arbor, Michigan, USA; University at Buffalo

**Keywords:** *Plasmodium* ortholog, *Toxoplasma gondii*, cell attachment, cell invasion, micronemes

## Abstract

*Toxoplasma gondii* and its *Plasmodium* kin share a well-conserved invasion process, including sequential secretion of adhesive molecules for host cell attachment and invasion. However, only a few orthologs have been shown to be important for efficient invasion by both genera. Bioinformatic screening to uncover potential new players in invasion identified a previously unrecognized *T. gondii* ortholog of *Plasmodium* glycosylphosphatidylinositol-anchored micronemal antigen (TgGAMA). We show that TgGAMA localizes to the micronemes and is processed into several proteolytic products within the parasite prior to secretion onto the parasite surface during invasion. TgGAMA from parasite lysate bound to several different host cell types *in vitro*, suggesting a role in parasite attachment. Consistent with this function, tetracycline-regulatable TgGAMA and TgGAMA knockout strains showed significant reductions in host cell invasion at the attachment step, with no defects in any of the other stages of the parasite lytic cycle. Together, the results of this work reveal a new conserved component of the adhesive repertoire of apicomplexan parasites.

**IMPORTANCE** *Toxoplasma gondii* is a successful human pathogen in the same phylum as malaria-causing *Plasmodium* parasites. Invasion of a host cell is an essential process that begins with secretion of adhesive proteins onto the parasite surface for attachment and subsequent penetration of the host cell. Conserved invasion proteins likely play roles that were maintained through the divergence of these parasites. Here, we identify a new conserved invasion protein called glycosylphosphatidylinositol-anchored micronemal antigen (GAMA). Tachyzoites lacking TgGAMA were partially impaired in parasite attachment and invasion of host cells, yielding the first genetic evidence of a specific role in parasite entry into host cells. These findings widen our appreciation of the repertoire of conserved proteins that apicomplexan parasites employ for cell invasion.

## Abstract (precis)

*Toxoplasma gondii* is a successful human pathogen in the same phylum as malaria-causing *Plasmodium* parasites. Invasion of a host cell is an essential process that begins with secretion of adhesive proteins onto the parasite surface for attachment and subsequent penetration of the host cell. Conserved invasion proteins likely play roles that were maintained through the divergence of these parasites. Here, we identify a new conserved invasion protein called glycosylphosphatidylinositol-anchored micronemal antigen (GAMA). Tachyzoites lacking TgGAMA were partially impaired in parasite attachment and invasion of host cells, yielding the first genetic evidence of a specific role in parasite entry into host cells. These findings widen our appreciation of the repertoire of conserved proteins that apicomplexan parasites employ for cell invasion.

## INTRODUCTION

Parasitic infections persist as a global concern, resulting in significant human and animal diseases along with financial impacts on health care and agriculture. Among the most successful and infectious parasites are two members of the phylum *Apicomplexa*, *Toxoplasma gondii* and *Plasmodium* species. *T. gondii* infects approximately one-third of the global human population (1), while *Plasmodium* parasites, which cause malaria, infect over 200 million people and result in over half a million deaths yearly (2). As obligate intracellular parasites, they share conserved subcellular structures and organelles, an essential invasion process, and several steps of the lytic cycle. Therefore, understanding the functions of conserved proteins in the biology of one parasite is often relevant to the other parasite, and the systems are considered to be highly complementary. *T. gondii* generally remains the more genetically tractable of the two parasites, and its wider host range permits the use of mice as a suitable experimental model of infection.

Invasion of host cells is an essential process for propagation of the apicomplexan lytic cycle. While a large number of molecules from the micronemes and rhoptries have been characterized, only a few have been shown to play conserved and critical roles in the invasion process, such as RON2, AMA1, and MIC2/TRAP (3–7). Recent advances in genetic manipulation tools for *T. gondii*, such as conditional Cre-mediated recombination (8) and CRISPR-CAS9 (9), have shown that, despite being important, previously designated “essential” genes can be disrupted and parasites are viable, albeit substantially compromised. Although compensatory effects due to gene knockout are coming to light, it also remains possible that additional as-yet-undiscovered proteins contribute to invasion in the absence of known key players.

Glycosylphosphatidylinositol (GPI)-anchored micronemal antigen (GAMA) was identified in *Plasmodium falciparum* while searching for putative invasion proteins that are conserved between merozoites and sporozoites (10). Although a knockout of *P. falciparum* GAMA (PfGAMA) has not been reported, PfGAMA was shown to bind to erythrocytes, and anti-PfGAMA antibodies inhibited merozoite invasion, suggesting an adhesive role for PfGAMA (10, 11). A *T. gondii* protein with similarity to PfGAMA was identified in a proteomic study of secretory products (12) and a small screen for genes potentially involved in invasion (13), but it was not fully recognized as an ortholog of PfGAMA in those previous studies. In this study, we reidentified *T. gondii* GAMA (TgGAMA) in a larger bioinformatic screen and further defined its role in *T. gondii* infection, revealing a phylogenetically conserved role in parasite attachment and invasion.

## RESULTS

### Identification of apicomplexan-specific invasion-related proteins, including TgGAMA.

**FIG 1:**
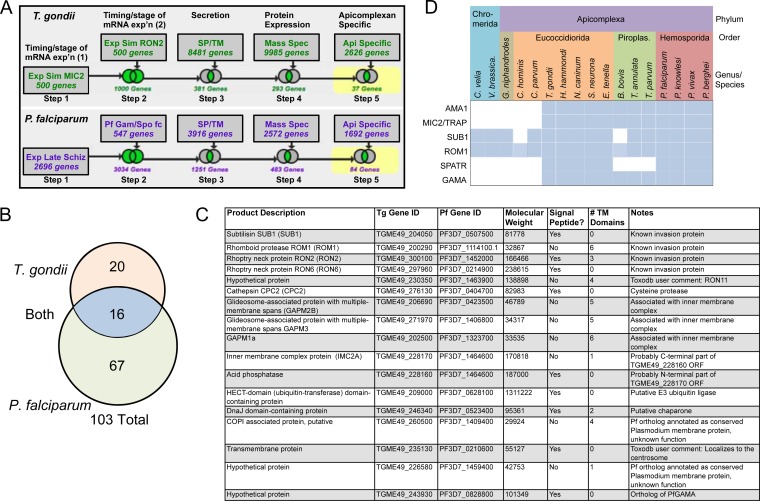
Identification of apicomplexan-specific putative invasion proteins. (A) Schematic representation of the search strategies used to find *T. gondii* (top row) and *P. falciparum* (bottom row) invasion proteins in the *Toxoplasma* and *Plasmodium* genome databases. Exp or exp’n, expression; Sim, simulation; SP, signal peptide; TM, transmembrane; Spec, spectroscopy; Api, apicomplexan; Schiz, schizont; gam, gametocyte; Spo, sporozoites; fc, fold change. (B) Venn diagram depicting the number of proteins identified exclusively in the *T. gondii* search (top), in both the *T. gondii* and *P. falciparum* searches (middle), and exclusively in the *P. falciparum* search (bottom). Note that two of the hits (TGME49_228170 and TGME49_22180) identified in both the *T. gondii* and *P. falciparum* searches likely correspond to one gene; thus, 16 proteins are depicted in the middle section rather than 17. (C) Features of the hits identified in the *T. gondii* and *P. falciparum* searches. (D) Phylogenetic distribution of microneme proteins that are conserved among most apicomplexans.

Secretory proteins involved in cell invasion by apicomplexan parasites have a series of features in common. For example, the expression of such proteins is coregulated, coinciding with the biogenesis of apical micronemes and rhoptries during mitosis and cytokinesis in *T. gondii* and during the formation of daughter merozoites, a process termed schizogony, in *P. falciparum*. Secretory proteins also typically possess a signal peptide and/or one or more transmembrane domains for membrane anchorage. We used these features, along with additional filters for protein expression (mass spectroscopy/proteomics) and presence in both *T. gondii* and *P. falciparum*, but not mammals, to search the genome databases for novel apicomplexan-specific putative invasion proteins (Fig. 1A). These searches identified 103 proteins, including 20 yielded by the *T. gondii* search, 67 identified by the *P. falciparum* search, and 16 that were identified in both searches (Fig. 1B; see Table S1 in the supplemental material). Nine of the 16 proteins identified in both searches have been characterized in invasion (SUB1, ROM1, RON2, RON6, and RON11) (14–20) or are implicated in invasion by their association with the inner membrane complex (GAPM2B, GAPM3, GAPM1a, and IMC2A). The remaining seven proteins include a putative E3 ubiquitin ligase, a putative chaperone, and several hypothetical proteins. We noted that one of the proteins (TGME49_243930) annotated as hypothetical in *T. gondii* was similar to PfGAMA (BLASTp E value of 3e-16, 48% coverage, 23% identity). Likenesses included sections of strong amino acid sequence identity, predicted secondary structure dominated by alpha helices, well-conserved cysteine residues suggestive of disulfide bonds, and a putative GPI signal sequence (see Fig. S1A in the supplemental material). BLAST searches of the EuPathDB database (eupathdb.org) indicated that GAMA is conserved in parasites that fully commit to intracellular residence, including *Plasmodium*, *Babesia*, *Theileria*, *Eimeria*, and *T. gondii*, but not *Cryptosporidium* or *Gregarina*, which occupy extracytoplasmic niches (Fig. 1D; see Tables S1 and S2 in the supplemental material). This pattern is identical to that of other apicomplexan-specific microneme proteins, including AMA1 and MIC2/TRAP, and is similar to the sporozoite protein with altered thrombospondin repeats (21–23). GAMA is also not found in the chromerids (*Chromera velia* or *Vitrella Brassica*), which are free-living photosynthetic relatives of the phylum *Apicomplexa*. Together, these findings suggest that GAMA was acquired or evolved relatively late in the advancement of intracellular parasitism. Because of these intriguing features, we chose to further investigate the function TgGAMA in the infection biology of *T. gondii*.

10.1128/mSphere.00012-16.4 Table S1  Proteins identified in this study. Download Table S1, XLSX file, 0.02 MB. Copyright © 2016 Huynh and Carruthers. 2016 Huynh and Carruthers This content is distributed under the terms of the Creative Commons Attribution 4.0 International license.

10.1128/mSphere.00012-16.5 Table S2  GAMA orthologs identified in apicomplexans in this study. Download Table S2, DOCX file, 0.1 MB. Copyright © 2016 Huynh and Carruthers. 2016 Huynh and Carruthers This content is distributed under the terms of the Creative Commons Attribution 4.0 International license.

10.1128/mSphere.00012-16.1 Figure S1  Multiple-sequence alignments of apicomplexan GAMA orthologs. Shown are *T. gondii* GAMA (NCBI reference sequence XP_002366882), *N. caninum* GAMA (CBZ52063.1), *P. yoelii* GAMA (XP_727931.1), *P. falciparum* GAMA (XP_001349238.1), and *P. vivax* GAMA (XP_001614875.1). Sequence alignment was created with MultAlin. Secondary-structure elements (helixes, cylinder; β-sheet, arrow) were predicted by JPred. Perfectly conserved positions are boxed with a black outline and shaded yellow. Cysteine residues are boxed with a black outline and shaded green. Download Figure S1, PDF file, 0.2 MB. Copyright © 2016 Huynh and Carruthers. 2016 Huynh and Carruthers This content is distributed under the terms of the Creative Commons Attribution 4.0 International license.

### TgGAMA is a multidomain and multiply processed protein.

**FIG 2:**
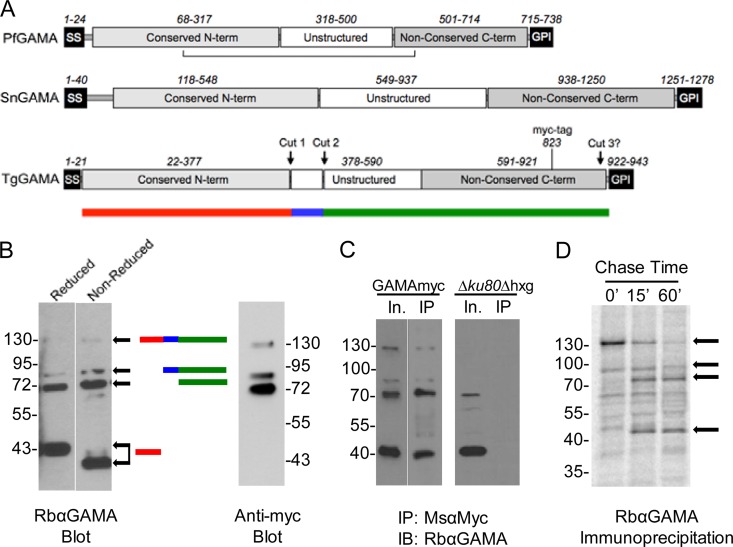
Domain organization of GAMA proteins and proteolytic processing of TgGAMA. (A) Schematic representations of PfGAMA, SnGAMA, and TgGAMA. Numbers depict approximate amino acid positions of major domains or elements. The horizontal line below PfGAMA indicates an intradomain disulfide bond. Approximate proteolytic processing sites are shown for TgGAMA. The position of a myc tag upstream of the GPI anchor at amino acid 823 in TgGAMA is shown. The colored bar below TgGAMA symbolizes junctions of the processing sites for panel B. term, terminus. (B) Western blot assay (left) of reduced and nonreduced parasite lysates with color bars on the right indicating the predicted fragments. Western blot assay (right) probed with anti-Myc antibody recognizes only the full-length protein and C-terminal fragments. (C) Parasite lysate input (In.) immunoprecipitated (IP) with mouse anti-Myc antibody and probed with rabbit anti-GAMA antibody (GAMAmyc lysate, left blot; Δ*ku80* lysate, right blot). IB, immunoblot. (D) ^35^S metabolic labeling, followed by immunoprecipitation with anti-GAMA. Arrows indicate GAMA-specific bands based on Western blotting patterns. The values beside the blots in panels B to D are molecular sizes in kilodaltons.

We created schematic representations of PfGAMA, *Sarcocystis neurona* GAMA (SnGAMA) and TgGAMA on the basis of reported features of PfGAMA (10) and multiple-sequence alignments (Fig. 2A; see Fig. S1 in the supplemental material). The general elements include a predicted signal sequence, a conserved N-terminal domain, and a nonconserved central unstructured region. Apicomplexan GAMA proteins also feature a nonconserved C-terminal domain and a GPI signal addition sequence. No significant matches to any known domains, adhesive or otherwise, were identified by Pfam or MotifScan. Hinds et al. (10) identified the approximate domain boundaries and processing sites of PfGAMA and showed that the N- and C-terminal domains are held together by a disulfide bond. Using a rabbit antibody generated against recombinant full-length TgGAMA, we showed that TgGAMA is also processed into multiple fragments within the parasite, including an ~130-kDa full-length species and major processed products of 72 and 43 kDa (Fig. 2B, left panel, reduced lane). To identify bands derived from the C-terminal fragment, we expressed a copy of TgGAMA containing a myc epitope tag at amino acid position 823 in a strain termed GAMAmyc. Anti-Myc antibody reacted with the 130-kDa precursor species and the 72-kDa product but not with the 43-kDa product. Absence of reactivity with the 43-kDa species identifies it as the N-terminal fragment (Fig. 2B, right panel). The lack of major upward shifts in the pattern of bands under nonreduced conditions suggests that, unlike PfGAMA, TgGAMA lacks an interdomain disulfide bond. The N-terminal fragment did, however, migrate moderately faster under nonreduced conditions, implying that the conserved cysteines in this domain form disulfide bonds that maintain globularity and increase its electrophoretic mobility in the gel. Despite the absence of an interdomain disulfide bond, the fragments remain associated with each other in the parasite, as shown by the immunoprecipitation of all of the fragments with anti-Myc antibodies (Fig. 2C). To better understand the proteolytic processing of TgGAMA, we performed a metabolic labeling pulse-chase immunoprecipitation experiment, which revealed that processing of the full-length TgGAMA occurred within 15 min of synthesis (Fig. 2D). An ~90-kDa minor species observed by Western blotting was present during the labeling period and in the 15-min chase sample but decreased in the 60-min sample, suggesting that it is an intermediate product. Altogether, these findings imply that TgGAMA is synthesized as a precursor species that is proteolytically processed via an intermediate to two major fragments. On the basis of the size of the products, we estimate that processing occurs at two sites within the central unstructured region (Fig. 2A). A third processing site near the putative GPI anchor likely exists because we observed soluble TgGAMA secreted from the parasite (see below).

### TgGAMA is a microneme-derived adhesive protein.

**FIG 3:**
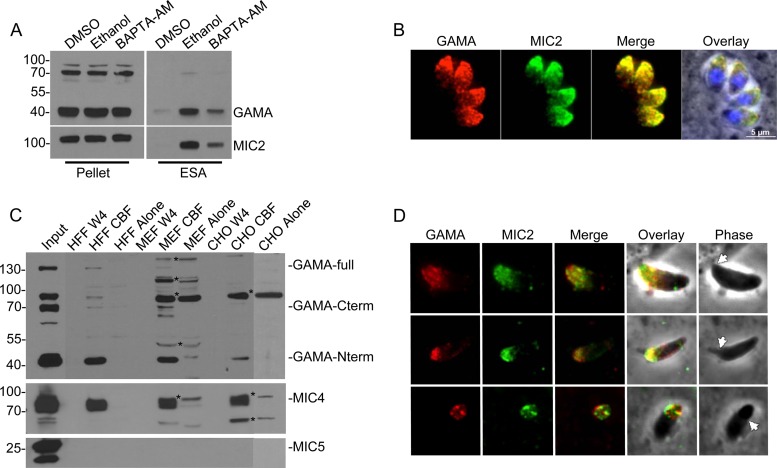
TgGAMA is a secreted micronemal protein capable of binding host cells. (A) Parasite lysate (pellet) and induced ESA fractions. ESA fractions were collected from culture supernatants after parasites were treated for 2 min at 37°C with 1% DMSO, 1% ethanol, or 1 µM BAPTA-AM. (B) Indirect immunofluorescence assay of intracellular parasites with rabbit anti-GAMA and mouse anti-MIC2 antibodies. (C) Western blot assays of material from cell binding assays. Parasite sonicate was incubated with host cells (HFF, MEF, or CHO cells), lysed in RIPA buffer, and resolved by 10% SDS-PAGE. Blots were probed with rabbit anti-GAMA, rabbit anti-MIC4, or rabbit anti-MIC5 antibodies. W4, wash 4; GAMA-full, full-length unprocessed GAMA. Asterisks indicate nonspecific bands. (D) TgGAMA mobilizes to the surface of invading parasites (top) and is capped posteriorly along with MIC2 as the tachyzoite penetrates the host cell (middle and bottom). Arrows indicate the moving junction. The values beside the blots in panels A and C are molecular sizes in kilodaltons.

Microneme secretion is dependent upon an increase in the parasite cytosolic calcium concentration (24), which can be stimulated by treatment with 1% ethanol (25). Consistent with secretion from the micronemes, ethanol treatment increased TgGAMA abundance in the excreted-secreted antigen (ESA) fraction, an effect that was partially reversed by cotreatment with the calcium chelator 1,2-bis-(2-aminophenoxy)ethane-*N*,*N*,*N*ˈ,*N*ˈ-tetraacetic acid tetra(acetoxymethyl) ester (BAPTA-AM) (Fig. 3A). Immunofluorescence staining of intracellular replicating parasites showed that TgGAMA colocalizes with a known micronemal protein, MIC2 (Fig. 3B), confirming that TgGAMA is secreted from the micronemes.

Several microneme proteins, including MIC2 and MIC4, have been characterized as adhesive proteins on the basis of their ability to bind host cells *in vitro* (26, 27). To assess the adhesive properties of TgGAMA, we incubated a sonicate of GAMAmyc parasites with human foreskin fibroblasts (HFF), mouse embryonic fibroblasts (MEF), or Chinese hamster ovary (CHO) cells. After washing unbound protein, we measured TgGAMA in the cell-bound fraction (CBF) by Western blotting. TgGAMA was detected in the CBF of all of the host cells tested (Fig. 3C), mirroring the cell binding observed with MIC4. In contrast, no binding by a nonadhesive microneme protein, MIC5, was seen.

Microneme proteins that function in host cell attachment are often secreted onto the apical surface of an invading tachyzoite before being capped toward the posterior end as the parasite penetrates the host cell (28). To assess the behavior of TgGAMA during *Toxoplasma* invasion, we fixed HFF cells that were briefly exposed to GAMAmyc parasites and stained for TgGAMA without permeabilizing the parasite to visualize surface-exposed antigens. As shown in Fig. 3D, TgGAMA is secreted onto the apical surface of attached parasites, where it colocalized with MIC2. TgGAMA is capped toward the posterior end of the parasite during penetration of the target cell, which is also similar to MIC2 and a hallmark of micronemal adhesive proteins. Altogether, these findings suggest that invading parasites secrete TgGAMA from the micronemes onto the apical surface for potential interaction with host receptors involved in parasite entry into host cells.

### iGAMA expression is efficiently downregulated upon ATc treatment.

**FIG 4:**
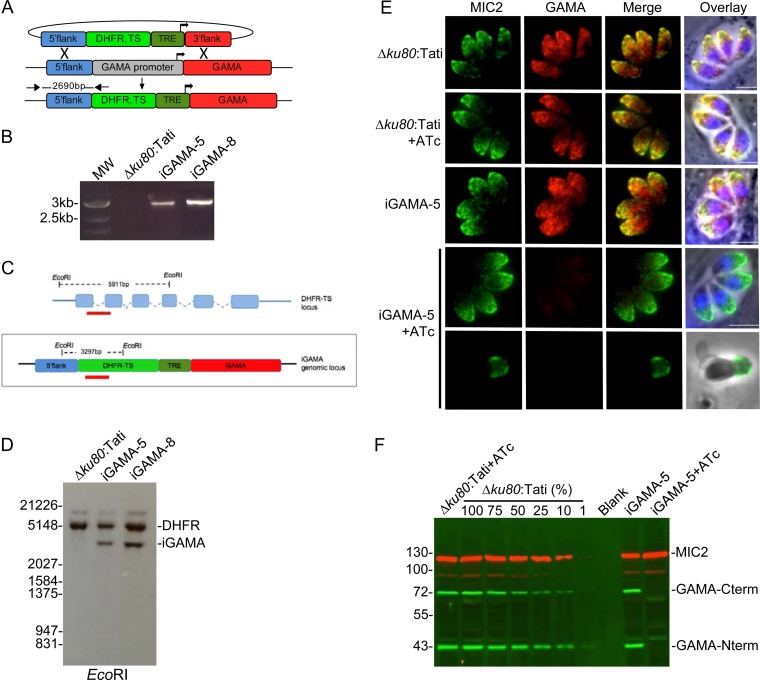
Establishment of a TgGAMA conditional-knockdown strain. (A) Schematic strategy used to generate iGAMA by replacement of the endogenous promoter with TRE and a truncated SAG4 promoter by double homologous recombination. (B) PCR assay confirming integration of the DHFR-TS selectable marker and TRE-SAG4 (T7S4) cassette at the GAMA gene locus. The primers used are indicated in panel A. MW, molecular weight. (C) Schematic showing EcoRI restriction sites in the endogenous DHFR-TS locus and the iGAMA locus used for Southern blot analysis. Predicted fragments for the endogenous DHFR locus and the iGAMA locus are shown. Annealing sites for the probe are indicated by red bars. (D) Southern blot analysis of iGAMA clones demonstrates that the T7S4 cassette was integrated as a single copy into the genome. Bands from the endogenous DHFR-TS locus and the iGAMA locus are indicated. Values on the left indicate molecular weight in base pairs. (E) Immunofluorescence imaging shows downregulation of TgGAMA following 24 h of treatment with ATc, indicated by the absence of microneme staining with rabbit anti-GAMA antibody. ATc has no effect on TgGAMA expression in Δ*ku80*:Tati parental tachyzoites. The bottom row shows lack of surface staining for an invading iGAMA parasite after ATc treatment, further confirming the specificity of the antibody. Bars, 5 µm. (F) Infrared fluorescence Western blot assay including a standard curve of parental parasite lysates from 100% to 1% shows that the level of TgGAMA expression (green bands) in iGAMA tachyzoites is similar to that in WT parasites and that TgGAMA expression is reduced to less than 1% of the WT level after 48 h of ATc treatment. Staining for MIC2 (red bands) was included as a loading control. Values on the left indicate molecular mass in kilodaltons.

Since multiple attempts to directly disrupt *TgGAMA* in RH parasites or genetically amenable RHΔ*ku80* parasites failed, we sought to generate a conditional-knockdown strain wherein expression could be turned off by the addition of anhydrotetracycline (ATc). A cassette (T7S4) consisting of the dihydrofolate reductase-thymidylate synthase (DHFR-TS) selectable marker and Tet-regulatable elements (TRE) composed of seven tandem TetO sequences and a truncated SAG4 promoter was used to replace the endogenous *TgGAMA* promoter by homologous recombination (Fig. 4A). PCR analysis confirmed integration of the selectable marker into the *TgGAMA* promoter region in two independent clones, iGAMA-5 and iGAMA-8 (Fig. 4B). Southern blotting with a probe against a fragment of the DHFR-TS marker, which recognizes both the endogenous DHFR-TS locus and the T7S4 cassette (Fig. 4C), demonstrated that a single copy of the T7S4 cassette was integrated into the genome (Fig. 4D). Immunofluorescence analysis showed normal microneme expression of TgGAMA in untreated iGAMA-5 parasites, whereas TgGAMA was undetectable after treatment with ATc in intracellular or invading parasites (Fig. 4E). Western blot analysis showed that 48 h of treatment with ATc downregulated TgGAMA expression to less than 1% of the normal expression level on the basis of comparison to a dilution series of parental strain lysate (Fig. 4F). iGAMA-5 parasites without ATc treatment showed levels of TgGAMA expression similar to those of parental parasites, indicating that the T7S4 and TgGAMA promoters are similar in strength. Identical results were obtained with both iGAMA-5 and iGAMA-8 clones; therefore, all subsequent assays were carried out with iGAMA-5 alone, which is referred to here as iGAMA.

### TgGAMA knockdown parasites show normal plaque formation and replication.

As a first pass to identify defects due to the absence of TgGAMA, we performed plaque assays to examine the entire lytic cycle. iGAMA parasites treated with ATc showed the same density and size of plaques as untreated or parental parasites (see Fig. S3A and B in the supplemental material). To investigate growth on a shorter time scale of two or three cell divisions, we determined the number of parasites per intracellular vacuole at 18 h postinfection. Replication of iGAMA treated with ATc was indistinguishable from that of parental parasites (see Fig. S3C).

10.1128/mSphere.00012-16.2 Figure S2  Parameters and settings used for bioinformatic searches to identify apicomplexan-specific putative invasion proteins. The search parameters used for the *Toxoplasma* genome database (ToxoDB) are shown on the left, and those used for the *Plasmodium* genome database are shown on the right. Parameters are shown as screen shots from the respective database websites. Download Figure S2, PDF file, 0.4 MB. Copyright © 2016 Huynh and Carruthers. 2016 Huynh and Carruthers This content is distributed under the terms of the Creative Commons Attribution 4.0 International license.

10.1128/mSphere.00012-16.3 Figure S3  TgGAMA does not play a role in growth and replication. (A) iGAMA + ATc parasites form normal plaques after 7 days of growth on HFF cells. (B) Quantification of plaque size by ImageJ indicates no significant differences between parental and iGAMA+ATc tachyzoites. (C) Replication assay enumerating parasites per vacuole following 18 h of intracellular growth. ns, not significant. Download Figure S3, PDF file, 0.2 MB. Copyright © 2016 Huynh and Carruthers. 2016 Huynh and Carruthers This content is distributed under the terms of the Creative Commons Attribution 4.0 International license.

### TgGAMA is necessary for efficient and rapid invasion.

**FIG 5:**
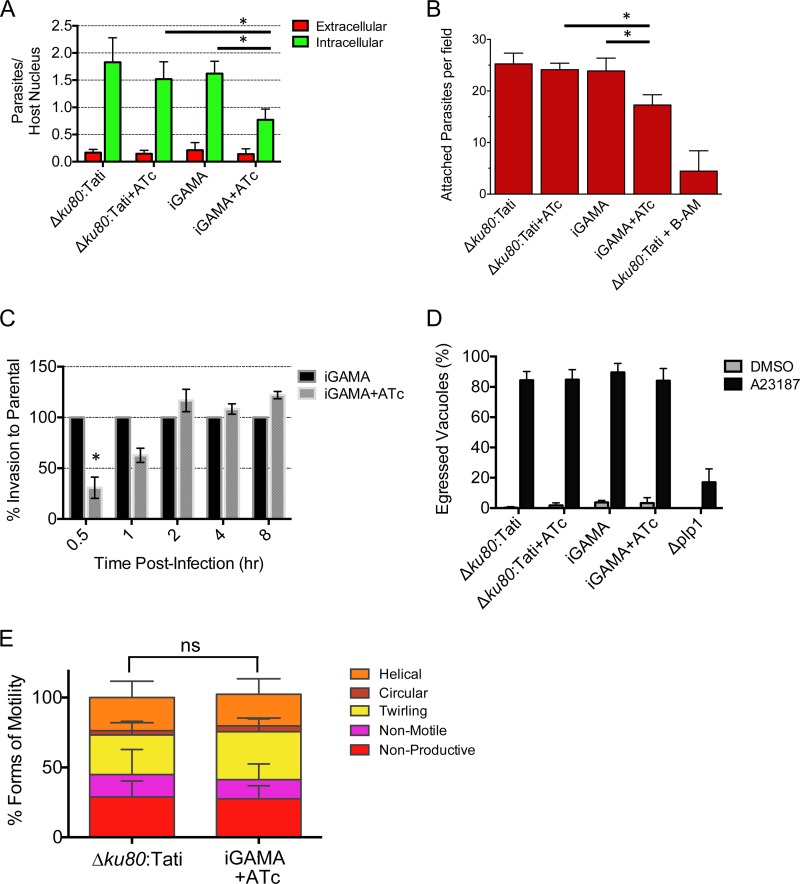
iGAMA knockdown parasites are defective in host cell attachment. (A) Red-green invasion assay of tachyzoites after 20 min of incubation with HFF cells. (B) Attachment of cytochalasin D-treated tachyzoites to HFF cells. Parasites were preloaded with calcein green AM and then incubated with cytochalasin D and allowed to attach to host cells for 15 min with cytochalasin D. BAPTA-AM-treated Δ*ku80*:Tati parasites are a negative control for attachment. (C) Timed invasion assay. Shown are the results of a red-green invasion assay of tachyzoites after 0.5, 1, 2, 4, or 8 h of incubation with HFF cells. (D) Induced-egress assay. Egress from vacuoles grown for 28 h in chamber slides was induced with the calcium ionophore A23187, and occupied versus unoccupied vacuoles were enumerated. (E) Modes of gliding motility. Parental and iGAMA+ATc tachyzoites with different types of gliding motility were enumerated by video microscopy. Data are the average ± the standard error of the mean of at least three independent experiments for all graphs. *, *P* < 0.05; ns, not significant.

Although the above results rule out major defects in the lytic cycle, it remained possible that TgGAMA contributes to an individual step in the cycle. To examine its role in cell entry, we performed invasion experiments that utilized differential red-green staining to identify extracellular/attached parasites and intracellular/invaded parasites, respectively. Compared to the parental Δ*ku8*0:Tati strain with or without ATc treatment, iGAMA with ATc treatment showed a statistically significant 50% decrease in invasion (Fig. 5A). A reduction in tachyzoite invasion can be due to a defect in initial attachment to the target cell or penetration of the cell. To determine if TgGAMA contributes to attachment, cytochalasin D-treated parasites were inoculated onto HFF cells, allowing attachment to but not penetration of host cells. As shown in Fig. 5B, iGAMA+ATc tachyzoites showed a significant defect in attachment compared to untreated parasites and to parental tachyzoites with or without ATc. This, along with surface localization during invasion and cell binding, suggests that TgGAMA is an adhesin that facilitates *Toxoplasma* attachment to target cells.

The red-green staining assay allows invasion for 20 min, but it has been shown that, given a longer time for invasion, several other genetically disrupted strains can recover to nearly parental strain levels (5, 23). As shown in Fig. 5C, iGAMA tachyzoites also recovered normal invasion levels when given additional time for infection. More specifically, whereas iGAMA parasites showed an invasion deficiency at 0.5 or 1 h postinfection (relative to the end of the 20-min invasion period), invasion reached normal levels at 2, 4, or 8 h postinfection. In determining whether other steps of the lytic cycle are affected, we assessed induced egress (Fig. 5D) and gliding motility by video microscopy (Fig. 5E), neither of which showed any differences between parental and iGAMA knockdown parasites. These results suggest that TgGAMA knockdown parasites are transiently deficient in cell invasion, thus providing an explanation for the lack of a plaque defect.

The invasion deficiency observed in the iGAMA strain could be the result of mislocalization of one of the other MIC proteins in addition to TgGAMA downregulation. Of the MIC proteins tested (MIC1, MIC2, MIC3, MIC4, MIC5, MIC6, MIC8, MIC10, MIC11, SUB1, PLP1, AMA1, and TLN4), all were correctly targeted to the micronemes (data not shown). The proper targeting of these MIC proteins reduces the likelihood that they are partners of TgGAMA, since MIC protein complexes are often dependent on the proper expression and trafficking of other members of the complex.

### GAMA knockout parasites recapitulate knockdown parasites.

**FIG 6:**
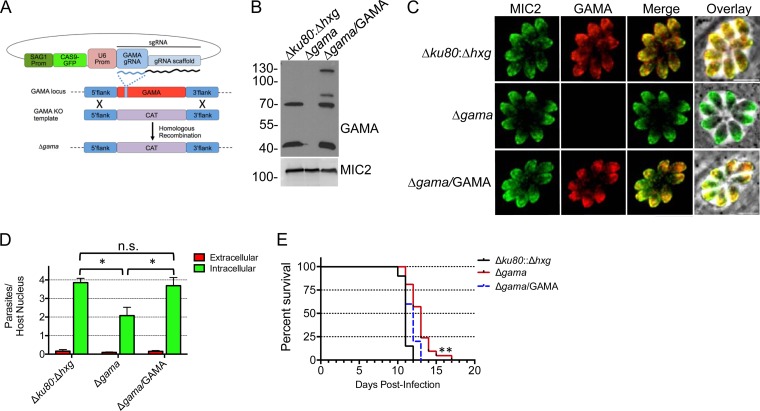
Generation of GAMA knockout (KO) by CRISPR-CAS9. (A) Schematic representation of CRISPR-CAS9-facilitated double homologous replacement of *TgGAMA* with a chloramphenicol acetyltransferase (CAT) selection cassette. Prom, promoter; sgRNA, single guide RNA. (B) Western blot analysis of parental, Δ*gama* mutant, and Δ*gama*/GAMA lysates. (C) Immunofluorescence confirmation of Δ*gama* and Δ*gama*/GAMA with rabbit anti-GAMA antibody. (D) Red-green invasion assay with HFF host cells. Data are the average ± the standard error of the mean of at least three independent experiments. *, *P* < 0.05; n.s., not significant (Student’s *t* test). (E) Δ*gama* mutant tachyzoites are modestly attenuated in virulence; Δ*gama*/GAMA tachyzoites show virulence kinetics similar to those of parental Δ*ku80*:Δ*hxg* tachyzoites. Mice were infected subcutaneously with 200 tachyzoites. The data represent infection of 20 mice each with Δ*ku80*:Δ*hxg* and Δ*gama* mutant tachyzoites and infection of 10 mice with Δ*gama*/GAMA tachyzoites. Concomitant plaque assays were performed to corroborate the numbers of viable tachyzoites injected. **, *P* < 0.0001 (Gehan-Breslow-Wilcoxon and Mantel-Cox tests). The values beside the blot in panel B are molecular sizes in kilodaltons.

CRISPR-CAS9 methodology was recently developed for use with *T. gondii*, making direct gene knockouts more feasible (9). A Δ*gama* mutant strain was generated by a single guide RNA (gRNA) and a TgGAMA PCR knockout construct in the Δ*ku80:*Δ*hxg* strain (Fig. 6A). The absence of the TgGAMA protein was confirmed by both Western blot (Fig. 6B) and immunofluorescence (Fig. 6C) analyses. The Δ*gama* mutant strain was complemented with full-length TgGAMA targeted to the “empty” Ku80 locus, with close-to-normal expression levels and processing (Fig. 6B) and proper localization to the micronemes (Fig. 6C). Assays of invasion by Δ*gama* mutant tachyzoites revealed a deficiency nearly identical to that observed in iGAMA tachyzoites (Fig. 6D). This confirmed that the efficient ATc-induced knockdown of TgGAMA was effectively equivalent to a knockout. A similar 50% reduction in the invasion of retinal pigmented epithelial cells by Δ*gama* mutant tachyzoites (data not shown) indicated that the invasion deficiency is not limited to one particular cell type. Reexpression of TgGAMA in the Δ*gama*/GAMA strain fully restored invasion efficiency to the level of the parental parasites (Fig. 6D).

A role for TgGAMA *in vivo* was evaluated in the mouse model of acute toxoplasmosis. iGAMA parasites showed no difference from parental parasites in the time to moribundity (data not shown). However, the Tati strain parasites are intrinsically attenuated (5, 29), thus confounding analysis of virulence in this background. For this reason, we used Δ*gama* mutant tachyzoites to reexamine the role of TgGAMA in virulence. Subcutaneous infection of CD-1 mice with 200 wild-type (WT) (Δ*ku80*:Δ*hxg*) or complemented (Δ*gama*/GAMA) tachyzoites was 100% fatal by day 13 postinfection (Fig. 6G), whereas mice infected with Δ*gama* mutant tachyzoites survived up to 4 days longer. This delayed virulence was statistically significant in both the Mantel-Cox and Gehan-Breslow-Wilcoxon tests, with *P* values of <0.0001.

## DISCUSSION

Since apicomplexan parasites are obligate intracellular organisms, attachment to and invasion of a host are fundamental and essential to their success. Accordingly, most apicomplexans have developed multiple strategies for invasion, including the evolution or acquisition of adhesive motifs that mimic those found on vertebrate host cells, recognition of a vast array of host receptors, and use of multiple invasion pathways. *Plasmodium* spp*.* and *Toxoplasma* had an ancestor in common several hundred million years ago. Although they encountered different hosts and environments through millions of years of divergence, these parasites have retained important conserved molecules in addition to expanding their repository of proteins to recognize specific host receptors on the specific cell types they preferentially invaded. *T. gondii* is notorious for its promiscuity, invading any nucleated cell, whereas *Plasmodium* parasites have narrowed their host cell range to erythrocytes and liver cells in their intermediate hosts (30, 31). The success of *T. gondii* may lie in its large repertoire of invasion-related proteins, such that secretion of this arsenal upon attachment provides the parasite with a multitude of options for host receptor binding. This might partially explain the relatively modest invasion defect observed upon the genetic disruption of numerous invasion proteins, save for a few more critical molecules, such as MIC2 and AMA1. The absence of one protein does not debilitate the parasite’s ability to attach to and invade the cell because of the presence of the other proteins. It is therefore of interest to identify conserved molecules since their retention versus modification or adaptation as these parasites diverged may imply a fundamental role in invasion.

The apical microneme organelles, along with rhoptries, are the origin of major players in invasion and are the logical focus in mining for additional molecular contributors to the process. A bioinformatic search strategy using ToxoDB, PlasmoDB, and EuPathDB was performed to identify other putative apicomplexan-specific secretory proteins that contribute to invasion. These novel components were identified by a stepwise search starting with the cell cycle timing of expression, followed by the presence of a signal peptide, indicating that the protein is secreted; protein expression based on the presence of mass spectrometry peptides; presence in both *P. falciparum* and *T. gondii* (and often other apicomplexans); and absence of orthologs in host cells. Although somewhat similar searches have been performed with one or the other parasite, to our knowledge, this is the first to extensively identify candidates that are conserved across most apicomplexans. Although candidates that were identified in both searches have the greatest potential to be authentic invasion proteins, hits that were identified in one or the other search should also be considered for follow-up studies. Coupling bioinformatic screens such as the one described here with future genome-wide screens that identify fitness coefficients for deletion of individual genes should be especially powerful for prioritizing mechanistic studies of key conserved invasion proteins.

To study the function of TgGAMA in tachyzoites, we took a genetic approach by generating both a direct knockout strain with CRISPR-CAS9 (9) and a tetracycline-regulated (32) iGAMA knockdown strain. There are advantages and disadvantages to both approaches. A benefit of iGAMA knockdown is that the role of TgGAMA is assessed within a short period (~48 h), thereby minimizing the likelihood of compensatory mechanisms such as upregulation of other MIC proteins that may occur during the selection of a direct knockout. However, iGAMA was created in the highly attenuated Tati strain, thus confounding assessment of TgGAMA as a virulence factor. We evaluated both strains and determined that they showed nearly identical invasion defects, indicating that a knockdown is effectively equivalent to a knockout. Although it remains unclear why we were unable to obtain a direct knockout with the initial multiple attempts in RH or RHΔ*ku80* parasites, a similar trend was seen for TgROM5, wherein conventional homologous recombination failed, yet subsequent cre/lox-based disruption yielded parasites with relatively mild phenotypes (17). These examples underscore the importance of using multiple genetic approaches and avoiding the tendency to ascribe importance or essentiality on the basis of unsuccessful disruption of a gene via a single approach.

TgGAMA demonstrated properties common to many MIC proteins that function in invasion, including being secreted onto the parasite surface upon attachment to a host cell, binding host cells *in vitro*, and contributing to efficient invasion. When invasion was limited to 20 min, iGAMA knockdown and Δ*gama* mutant parasites showed a significant reduction in invasion. However, given a longer incubation time, iGAMA knockdown parasites invaded to a level similar to that of parental parasites within 2 h, likely because of the presence of other adhesive molecules. This recovery of invasion efficiency provides a logical explanation for the absence of a plaque formation defect and likely contributes to the kinetics observed in mouse virulence assays, wherein mice infected with Δ*gama* mutant parasites showed a delay in the time to moribundity of up to 7 days. The combined data suggest that TgGAMA is an adhesive protein that plays a role during the initial attachment to the host cell but whose presence is not essential for invasion and that the secretion of other adhesins is capable of completing invasion. It is possible that TgGAMA has an unrealized partner protein that could also contribute to parasite attachment, and genetic disruption of both proteins may result in more severe phenotypes.

Structure-function studies are often carried out to determine the role or contribution of particular domains of a protein, as was done with PfGAMA (11). Erythrocyte binding activity was identified in the C-terminal domain of PfGAMA. Interestingly, alignment of several apicomplexan GAMA protein sequences indicated that the C-terminal domains of TgGAMA and PfGAMA are poorly conserved (see Fig. S1 in the supplemental material). This may reflect an adaptation of PfGAMA to an erythrocyte-specific receptor. Although the molecular identity of the erythrocyte receptor for PfGAMA remains unknown, the receptor was classified as being sialic acid independent on the basis of refractivity to neuraminidase treatment and sensitivity to chymotrypsin digestion (11). In contrast to the C-terminal domain, the GAMA N-terminal domain is well conserved among apicomplexan members of the protein family, suggesting a broader role in GAMA function. Disruption of *Plasmodium berghei* GAMA (PbGAMA, also termed PSOP9) showed marked defects in the mosquito and in initiation of mouse infection (33). More specifically, although ookinete development was normal in mosquitos infected with *PbGAMA* knockout parasites, 78% fewer oocysts and 99.7% fewer sporozoites were observed. Residual sporozoites were also incapable of establishing mouse infection when inoculated at doses equivalent to those of WT parasites. It remains to be seen whether the nonerythrocytic function(s) of *Plasmodium* GAMA is attributed to the conserved N-terminal domain and the extent that it involves engagement of receptors in the mosquito and mammalian hosts. Future studies of this kind provide insight into the evolution of the GAMA protein family and the contributions of its members to apicomplexan infections.

## MATERIALS AND METHODS

### Bioinformatics.

Identification of apicomplexan-specific putative invasion proteins from *T. gondii* was done by using a five-step strategy to search the *Toxoplasma* genome database (http://www.toxodb.org). For the specific parameters of each step, see Fig. S2 in the supplemental material. Briefly, step 1 identified genes with a cell cycle expression pattern similar to that of MIC2 (TGME49_201780). Step 2, which was added to step 1, identified genes with a cell cycle expression pattern similar to that of RON2 (TGME49_300100). Step 3, which intersected with the sum of steps 1 and 2, consisted of two substeps added together. Substep 1 identified all proteins with a signal peptide, whereas substep 2 identified proteins with one to seven transmembrane domains. Step 4, which intersected with the previous steps, identified proteins with evidence of expression based on mass spectrometry evidence. Step 5, which also intersected with the previous steps, identified proteins produced by *T. gondii* and *P. falciparum* but not by mammals. Identification of proteins from *P. falciparum* was performed by using a similar five-step strategy to search the *Plasmodium* genome database (http://www.plasmodb.org). All of the steps were the same except steps 1 and 2. Step 1 consisted of identifying proteins that were upregulated during late schizogony (32 to 40 h postinvasion) or in merozoites (40 to 5 h postinvasion) to yield proteins possibly involved in merozoite invasion. Step 2 identified proteins upregulated in sporozoites relative to gametocytes to survey proteins potentially involved in sporozoite invasion. A multiple-sequence alignment of GAMA orthologs was generated with MultAlin (http://multalin.toulouse.inra.fr/multalin/). Secondary-structure predictions were made with JPred 4 (http://www.compbio.dundee.ac.uk/jpred/). Signal sequence cleavage sites were identified with the SignalP 4.1 server (http://www.cbs.dtu.dk/services/SignalP/).

### Parasite culture, transfection, and selection.

*T. gondii* tachyzoites were maintained by growth in monolayers of HFF in Dulbecco’s modified Eagle’s medium (DMEM) containing 10% fetal bovine serum (FBS; Gibco), 2 mM glutamine, and 10 mM HEPES (D10 complete). The TatiΔ*Tgku80* strain (32) was transfected with a T7S4GAMA promoter replacement cassette based on pDT7S4myc (34). A fusion PCR construct consisting of a 2.2-kb fragment of the 5′ flank (*TgGAMA.5’2872960.F*, CTGTACATAATGCATTTGGGTTTGCGTGTAG; *dhfr_GAMA.-1000.R*, AAAATTGCGGGAAAGTCACGCATATCGGACCTGGAAGCCGCTGAC), a DHFR-TS selectable marker (*GAMA.−1000_dhfr.F*, GTCAGCGGCTTCCAGGTCCGATATGCGTGACTTTCCCGCAATTTT; *GAMA_T7S4.1.R*, GGGGAAGAAACTGACGCATTTCGATAGATCTGGTTGAAGACAGAC), and 3 kb of the GAMA coding sequence (CDS) with introns (*T7S4_GAMA.1.F*, GTCTGTCTTCAACCAGATCTATCGAAATGCGTCAGTTTCTTCCCC; *TgGAMA.2832.PacI.R*, GATCTTAATTAATTACAAAGCCGTCACAGCAACC) was amplified with nested primers *TgGAMA.5’2872715.R* (GATGGATAGAGAATTCCTCGATTACTGTCTAC) and *TgGAMA.1786.R* (TGATGCTGAGCACCTTGTTC).

For transfections, 1 × 10^7^ TatiΔ*Tgku80* strain parasites were electroporated with a Bio-Rad Gene Pulser II with 1.5-kV voltage and 25-µF capacitance settings and no resistance setting. Pyrimethamine selection was applied the day after transfection, and clones were isolated by limiting dilution in 96-well plates.

### Plasmids and constructs. (i) Myc-tagged GAMA construct.

The CDS of GAMA was amplified from a *T. gondii* cDNA library with primers TgGAMA.1.NsiI.F (GATCATGCATATGCGTCAGTTTCTTCCCCTTC) and TgGAMA.2832.PacI.R (GATCTTAATTAATTACAAAGCCGTCACAGCAACC). A C-myc tag was introduced by fusion PCR at amino acid 823 of the CDS with primers GAMA.aa823myc.F (AGTGAAACTGGAGAGGAACAAAAGTTGATTTCTGAAGAAGATTTGAAAGCGACGCTTTCT) and GAMA.aa823myc.R (AGAAAGCGTCGCTTTCAAATCTTCTTCAGAAATCAACTTTTGTTCCTCTCCAGTTTCACT). The myc-tagged GAMA CDS was introduced into the pM2AP.Ku80.HXG vector (23) by excising the existing M2AP CDS with NsiI and PacI.

### (ii) GAMA complementation construct.

The full-length GAMA CDS was amplified from a cDNA library and cloned into pM2AP.Ku80.HXGPRT as described for the Myc-tagged construct. This construct was electroporated into Δ*gama* mutant tachyzoites and selected with mycophenolic acid and xanthine, and clones were isolated by limiting dilution in 96-well plates.

### Rabbit GAMA antibody generation.

For polyclonal antibody production, recombinant His-tagged TgGAMA (a generous gift from M. Boulanger, University of Victoria, Victoria, British Columbia, Canada) was used for rabbit immunization, 0.25 mg per injection, with booster injections at 14, 42, and 56 days postinoculation, conducted by a commercial service (Thermo Scientific).

### Metabolic labeling and immunoprecipitation.

Pulse-chase labeling was performed as previously described (35), with the following modifications. We purified 2 × 10^8^/ml extracellular RH parasites in methionine- and cysteine-free DMEM supplemented with 1% FBS and labeled them with ^35^S-labeled methionine and cysteine (PerkinElmer) at 1 µCi/ml (final concentration) for 10 min in a 37°C heating block. The labeled parasites were then “chased” in DMEM growth medium containing 1% FBS, 5 mM methionine, and 5 mM cysteine for 0, 15, 30, 45, and 60 min (2 × 10^7^ tachyzoites per time point). Parasites collected at each time point were then centrifuged and lysed in radioimmunoprecipitation assay (RIPA) buffer plus protease inhibitors (Complete; Roche) and DNase/RNase and then immunoprecipitated with RbαGAMA antibody and protein G Sepharose. For brefeldin A treatment, brefeldin A was added at 10 µg/ml during the pulse and chase times and samples were collected at 0 and 60 min.

Following immunoprecipitation, samples were subjected to SDS-PAGE; the gels were then incubated in Amplify (Amersham) and dried in cellophane. Dried gels were exposed to a phosphorimaging screen and imaged on a Typhoon Imagine system.

### Indirect immunofluorescence microscopy.

All manipulations for indirect immunofluorescence microscopy were carried out at room temperature. Tachyzoite-infected HFF cells on eight-well chamber slides were fixed with 4% paraformaldehyde for 20 min and then washed in phosphate-buffered saline (PBS). Fixed cells were permeabilized with 0.1% Triton X-100 in PBS for 15 min, washed, and blocked in 10% FBS in PBS for 30 min. The wells were then stained with the primary antibodies (Msαmyc at 1:250 [9E10] DSHB, RbαMIC5 at 1:500, RbαMIC2 at 1:500, and RabαGAMA at 1:500), followed by Alexa 594- or Alexa 488-conjugated goat anti-mouse or anti-rabbit antibodies (Molecular Probes, Eugene, OR) and 4ˈ,6-diamidino-2-phenylindole. After washes in PBS–1% FBS–1% normal goat serum, slides were mounted in Mowiol and fluorescent images were collected at a total magnification of ×1,000 with a Zeiss Axio inverted microscope.

### ESA preparation and Western blotting.

ESA fractions were isolated as described previously (23). Briefly, filter-purified parasites were resuspended to a concentration of 5 × 10^8^/ml in DMEM–10 mM HEPES. Parasites were treated with 1% dimethyl sulfoxide (DMSO), 1% ethanol, or 1 µM BAPTA/AM (parasites were pretreated for 10 min) for 2 min at 37°C prior to placement on ice. Parasite lysate/pellet and ESA were separated by centrifugation at 1,000 × *g* for 10 min at 4°C and subjected to 10% SDS-PAGE. Gels were semidry electroblotted (Bio-Rad) onto polyvinylidene difluoride membranes. The antibodies used for Western blot analyses were rabbit anti-GAMA antibody at 1:10,000 (LI-COR; 1:30,000), rabbit anti-MIC4 antibody at 1:5,000, rabbit anti-MIC5 antibody at 1:5,000, and mouse anti-MIC2 antibody at 1:4,000 (LI-COR; 1:15,000). The secondary antibodies used for LI-COR Western blot assays were IRDye800 and IRDye680 (LI-COR).

### Cell binding.

Protein binding to host cells was assayed as described previously (23). Briefly, tachyzoite sonicate was prepared by resuspending 5 × 10^8^ tachyzoites in a mixture of 2 ml of PBS, 1 mM CaCl_2_, and 1 mM MgCl_2_ (PBS-CM) and sonicating them on ice with a Micronix microtip sonicator. Sonicates were centrifuged at 13,000 × *g* for 10 min at 4°C, added to confluent host cells (HFF, CHO cells, and MEF), and incubated at 12°C for 1 h. Cells were washed four times with PBS-CM and lysed with RIPA buffer. Sonicate, washes, and the CBF were separated by 10% SDS-PAGE and immunoblotted as described above.

### Southern blotting.

Southern blotting was performed as previously described (36), except for the following. The DHFR-TS probe was PCR amplified from pDT7S4myc (34) with primers *dhfrHXGPRTdhfr.F* (CAGCACGAAACCTTGCATTCAAACC) and *DHFR-TS.896.R* (GAATCCTTGTACTCTTCCTCCAGAAGG). Genomic DNA was digested with EcoRI, PvuII, and XmnI and run on a 1.5% agarose gel.

### Invasion and attachment.

Red-green invasion assays were performed as described previously, with the following modifications (37). Parasites were filter purified and resuspended to 5 × 10^7^/ml in DMEM–3% FBS–10 mM HEPES (D3). Calcein green AM (Invitrogen) was added to the parasites to a final concentration of 1 µM and incubated for 15 min with agitation in the dark. Parasites were then centrifuged at 1,000 × *g* and resuspended in fresh D3, and 1 × 10^7^ parasites were allowed to invade subconfluent host cell monolayers in eight-well chamber slides for 20 min before fixation. Slides were stained with anti-SAG1 antibodies and Alexa594 secondary antibodies to differentiate extracellular and intracellular parasites. For timed invasion assays, 2.5 × 10^5^ parasites were inoculated into each well of a chamber slide and the slide was fixed at a defined time point. For the cytochalasin D attachment assay, parasites were incubated with 1 µM calcein green AM (Life Technologies) for 20 min before the addition of 2 µM cytochalasin D for 10 min at room temperature. Parasites were centrifuged at 1,000 × *g* for 10 min, resuspended in D3 and 1 µM cytochalasin D, inoculated onto host cell monolayers, and allowed to attach for 20 min. Slides were fixed and enumerated on the basis of calcein green fluorescent parasites.

### Plaque and replication.

Parasites were inoculated into wells of a six-well plate and allowed to replicate undisturbed for 7 days. The wells were then stained with 0.2% cresol violet for 5 min and rinsed with double-distilled H_2_O. Images of the wells were scanned, and plaque number and size were analyzed with ImageJ.

For the replication assay, cells of an eight-well chamber slide were inoculated with 1.25 × 10^5^ tachyzoites and allowed to invade and grow for 18 h prior to fixation and indirect immunofluorescence assay.

### Induced egress.

Egress was induced as described previously (38). Briefly, parasites grown in HFF in the cells of an eight-well chamber slide for 28 h were treated with 1% DMSO or 2 µM calcium ionophore A23187 in assay buffer (Hanks’ balanced salt solution containing 1 mM CaCl_2_, 1 mM MgCl_2_, and 10 mM HEPES) and incubated for 2 min in a 37°C water bath. Egress was stopped by the addition of 2× fixative (8% formaldehyde in 2× PBS). Immunofluorescence analysis was performed with RabαSAG1 to identify parasites and MsαGRA7 to identify the parasitophorous vacuole membrane. At least 10 fields of view (total magnification, ×400) per condition were enumerated as occupied or unoccupied.

### Live gliding video microscopy.

Glass well dishes were coated with 50% FBS–50% PBS, incubated overnight at 4°C or for 1 h at 37°C, and washed with PBS before use. Parasites were filter purified and resuspended in HHE (Hanks’ balanced salt solution containing 10 mM HEPES, EDTA). Parasites were then added to coated dishes and allowed to settle for 5 min at room temperature. Dishes were then moved to a 37°C chamber with 5% CO_2_ and warmed for 5 min prior to the start of video recording. Each video consisted of 1 frame/s recorded for 90 s. Enumeration of the types of gliding motility was carried out by examining the videos in addition to maximum-projection images generated by the Zeiss AxioVision software.

### Design of gRNA for CAS9-mediated gene disruption.

Three TgGAMA gRNA constructs were generated by replacing the uracil phosphoribosyltransferase (UPRT) gRNA sequence in the pSAG1::CAS9-U6::sgUPRT plasmid (9) by QuikChange site-directed mutagenesis (GAMAgRNA-1, GAACAGAAACGGAAATATTC; GAMAgRNA-2, GTAGAAACCACTTTTTTGTG; GAMAgRNA-3, GTCGGGTTTCACAAATTATG).

### *In vivo* virulence.

Ten (for Δ*gama*/GAMA) or 20 (for Δ*ku80*:Δ*hxg* and Δ*gama*) 6-week-old female CD-1 mice were infected subcutaneously with 200 tachyzoites (in 200 µl of PBS). At the same time, 200 µl of each diluted parasite strain was inoculated into T25 flasks and incubated for 7 days before being stained with cresol violet to visualize plaques.
