# Supplementary material for: A Toxoplasma gondii Ortholog of Plasmodium GAMA Contributes to Parasite Attachment and Cell Invasion
Source: mSphere. 2016 Feb 10;1(1):e00012-16. doi: 10.1128/mSphere.00012-16 (PMC4863602; doi:10.1128/mSphere.00012-16)
Supplement: Table S2 [file sph001162017st5.docx]

| **Table S2. GAMA orthologs identified in apicomplexans** | | | |  |  |
| --- | --- | --- | --- | --- | --- |
| **[Gene ID]** | **[Score]** | **[E-Value]** | **[Organism]** | **[Molecular Weight]** | **[Protein Length]** |
| TGGT1_243930 | 654 | 0.00E+00 | T. gondii GT1 | 101323 | 943 |
| TGME49_243930 | 654 | 0.00E+00 | T. gondii ME49 | 101349 | 943 |
| TGVEG_243930 | 654 | 0.00E+00 | T. gondii VEG | 101353 | 943 |
| HHA_243930 | 618 | 0.00E+00 | H. hammondi strain H.H.34 | 101145 | 942 |
| NCLIV_018530 | 513 | 8.00E-174 | N. caninum Liverpool | 100599 | 955 |
| SN3_01400520 | 283 | 2.00E-85 | S. neurona SN3 | 136211 | 1278 |
| EMWEY_00053060 | 172 | 1.00E-46 | E. maxima Weybridge | 92901 | 883 |
| EfaB_MINUS_15349.g1307 | 172 | 6.00E-47 | E. falciformis Bayer Haberkorn 1970 | 83656 | 798 |
| EAH_00020390 | 148 | 3.00E-38 | E. acervulina Houghton | 89543 | 855 |
| ETH_00027620 | 100 | 5.00E-22 | E. tenella strain Houghton | 151300 | 1466 |
| ENH_00021490 | 98.2 | 2.00E-21 | E. necatrix Houghton | 70333 | 708 |
| PBANKA_070190 | 84 | 9.00E-17 | P. berghei ANKA | 71065 | 625 |
| PVX_088910 | 82.4 | 4.00E-16 | P. vivax Sal-1 | 82700 | 771 |
| PY07130 | 81.3 | 6.00E-16 | P. yoelii yoelii 17XNL | 70940 | 624 |
| PY17X_0702200 | 81.3 | 6.00E-16 | P. yoelii yoelii 17X | 70953 | 624 |
| PYYM_0702100 | 81.3 | 6.00E-16 | P. yoelii yoelii YM | 70953 | 624 |
| PF3D7_0828800 | 80.9 | 1.00E-15 | P. falciparum 3D7 | 85251 | 738 |
| PFIT_0831700 | 79.7 | 3.00E-15 | P. falciparum IT | 85658 | 742 |
| PCHAS_093610 | 78.6 | 6.00E-15 | P. chabaudi chabaudi | 70700 | 625 |
| PRCDC_0828100 | 76.3 | 3.00E-14 | P. reichenowi CDC | 83304 | 721 |
| PKH_050210 | 71.2 | 1.00E-12 | P. knowlesi strain H | 79680 | 722 |
| PCYB_051250 | 70.5 | 2.00E-12 | P. cynomolgi strain B | 76231 | 707 |
| BEWA_014050 | 45.1 | 4.00E-04 | T. equi strain WA | 71424 | 658 |
