# Supplementary figures and images for: A Toxoplasma gondii Ortholog of Plasmodium GAMA Contributes to Parasite Attachment and Cell Invasion
Source: mSphere. 2016 Feb 10;1(1):e00012-16. doi: 10.1128/mSphere.00012-16 (PMC4863602; doi:10.1128/mSphere.00012-16)

Figure S1

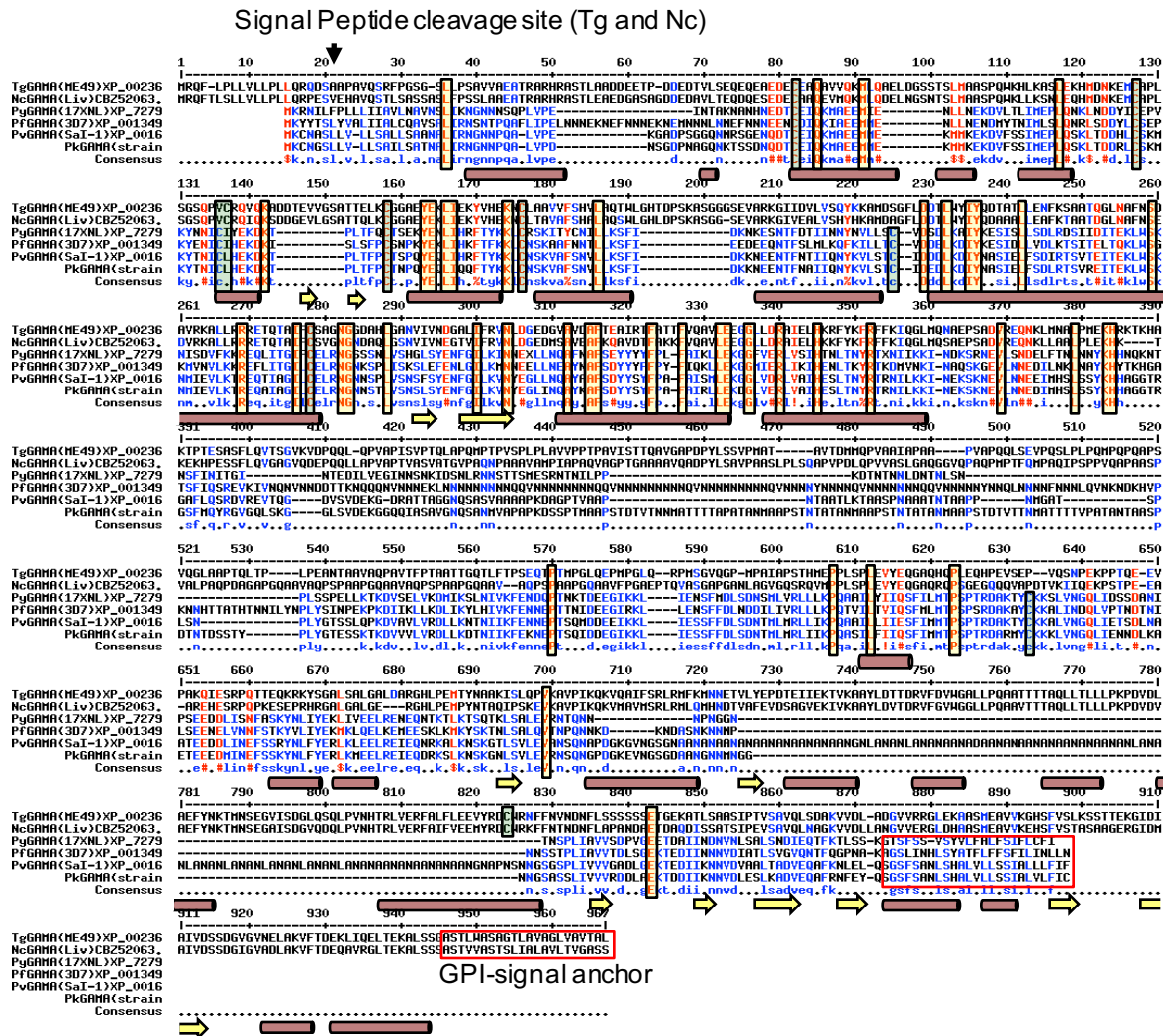

Supplement: Figure S1 [file sph001162017sf1.pdf]

Figure S3

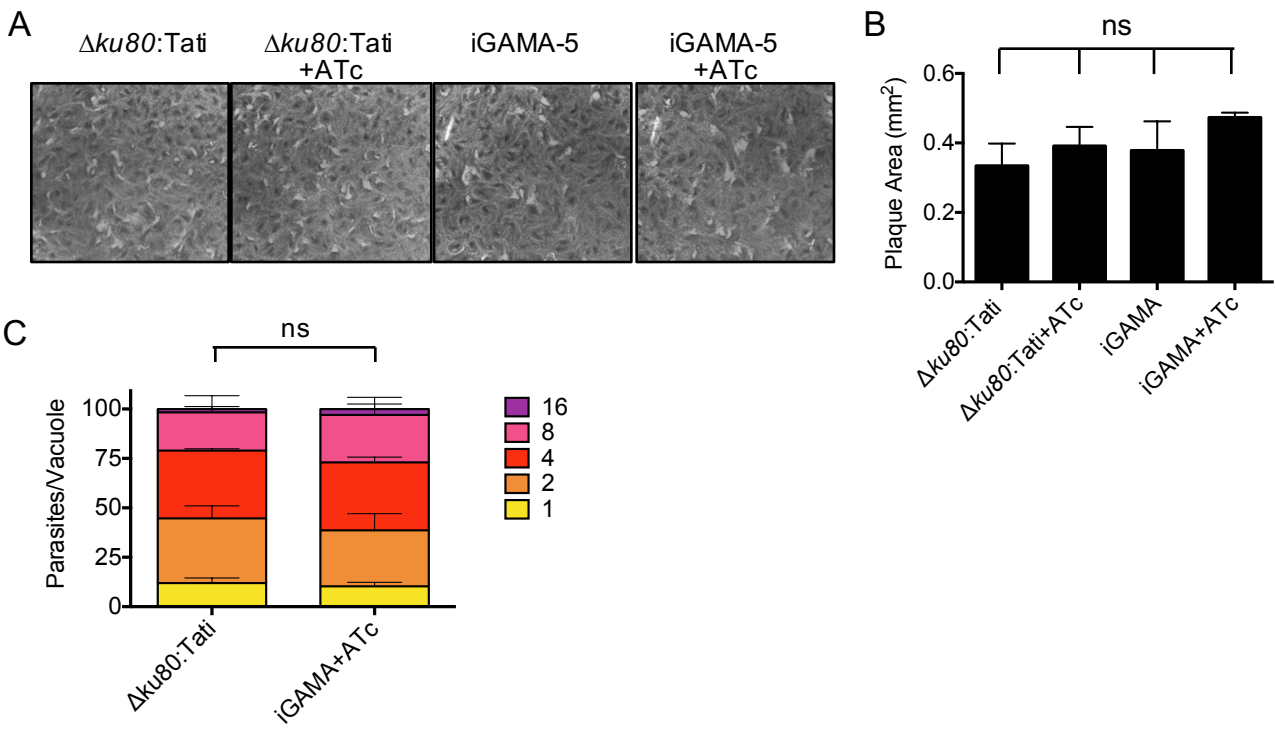

Supplement: Figure S3 [file sph001162017sf3.pdf]
