# Supplementary material for: A Toxoplasma gondii Ortholog of Plasmodium GAMA Contributes to Parasite Attachment and Cell Invasion
Source: mSphere. 2016 Feb 10;1(1):e00012-16. doi: 10.1128/mSphere.00012-16 (PMC4863602; doi:10.1128/mSphere.00012-16)
Supplement: Figure S2 [file sph001162017sf2.pdf]

Figure S2

Toxodb search

Step 1

STEP 1 : Exp Sim MIC2

Gene Id	TGME49_201780
Distance Method	Pearson Correlation
# of Matches	500
% of missing data points	10
Experiment	M.White Cell Cycle Microarray spline smoothed - Interpolated
Search Goal	Positive Correlation
Time Shift	0 hours

Results: 500 Genes

⊞ **Give this search a weight**

Step 2

STEP 2 : Exp Sim RON2

Gene Id	TGME49_300100
Distance Method	Pearson Correlation
# of Matches	500
% of missing data points	10
Experiment	M.White Cell Cycle Microarray spline smoothed - Interpolated
Search Goal	Positive Correlation
Time Shift	0 hours

Results: 500 Genes

⊞ **Give this search a weight**

Step 3

STEP 1 : Signal Pep

Organism	Toxoplasma gondii
Minimum SignalP-NN Conclusion Score	3
Minimum SignalP-NN D-Score	0.5
Minimum SignalP-HMM Signal Probability	0.5
Matches any or all advanced parameters	any

Results: 5867 Genes

⊞ **Give this search a weight**

STEP 2 : Transmb Dom

Organism	Toxoplasma gondii
Minimum Number of Transmembrane Domains	1
Maximum Number of Transmembrane Domains	7

Results: 4540 Genes

⊞ **Give this search a weight**

Step 4

STEP 4 : Mass Spec

Experiment/Samples : **Toxoplasma**, ***Toxoplasma gondii***, **Oocyst Partially Sporulated Proteome (VEG) (Possenti et al.)**, Oocyst proteome, **Oocyst proteome (M4 Typell) (Wastling)**, Oocyst peptides, **Oocyst proteome - Fractionated (M4 type II) (Fritz et al.)**, Oocyst Walls, Sporocysts/Sporozoites Cytosol, Sporocysts/Sporozoites Membrane, **Proteome During Infection in H. sapiens (ME49 VEG GT1 RH) (Wastling)**, peptides from 16 hour infection time course in Homo sapien host cells (ME49), peptides from 16 hour infection time course in Homo sapien host cells (VEG), peptides from 44 hour infection time course in Homo sapien host cells (ME49), peptides from 44 hour infection time course in Homo sapien host cells (RH), peptides from 44 hour infection time course in Homo sapien host cells (VEG), peptides from infection in Homo sapien host cells (GT1), **Tachyzoite Intra- and Extracellular Lysine-Acetylomes (RH) (Jeffers and Xue)**, extracellular acetylome, intracellular acetylome, **Tachyzoite Rhoptry proteome (RH) (Bradley et al.)**, purified rhoptries, **Tachyzoite conoid proteome (RH) (Hu et al.)**, conoid-depleted Fraction, conoid-enriched Fraction, **Tachyzoite membrane and cytosolic proteomes (RH) (Dybas et al.)**, cytosolic fraction by LC-MS/MS, 05/2007, membrane biotin-purified fraction by LC-MS/MS, 5/22/2007, membrane fraction by 1DE, LC-MS/MS, 10/2006, membrane fraction by 1DE, LC-MS/MS, 12/2006, membrane fraction by 2DE, LC-MS/MS, membrane fraction by LC-MS/MS, 2/3/2006, membrane fraction by LC-MS/MS, 5/10/2006, membrane fraction by LC-MS/MS, 5/2/2006, membrane fraction by LC-MS/MS, 6/2006, **Tachyzoite phosphoproteome - Calcium dependent (RH) (Nebl et al.)**, phosphopeptide-depleted (via Sequest), phosphopeptide-enriched (via Mascot), phosphopeptide-enriched (via Sequest), **Tachyzoite phosphoproteome from purified parasite or infected host cell (RH) (Treeck et al.)**, Infected host cell, phosphopeptide-depleted (peptide discovery against TgME49), Infected host cell, phosphopeptide-depleted (peptide discovery against TgGT1), Infected host cell, phosphopeptide-enriched (peptide discovery against TgME49), Infected host cell, phosphopeptide-enriched (peptide discovery against TgGT1), Purified tachyzoites phosphopeptide-depleted (peptide discovery against TgGT1), Purified tachyzoites phosphopeptide-depleted (peptide discovery against TgME49), Purified tachyzoites phosphopeptide-enriched (peptide discovery against TgGT1), Purified tachyzoites phosphopeptide-enriched (peptide discovery against TgME49), **Tachyzoite secretome (RH) (Zhou et al.)**, excreted/secreted antigen fraction by 2DE-MS, excreted/secreted antigen fraction by MudPIT, **Tachyzoite subcellular fractions (Moreno)**, sample A, sample G, **Tachyzoite total proteome (RH) (Wastling)**, Tris-insoluble fraction by 1DE, LC-MS/MS, Tris-insoluble fraction by MudPIT, Tris-soluble fraction by 1DE, LC-MS/MS, Tris-soluble fraction by MudPIT, total protein by 1DE, LC-MS/MS, total protein by 2DE-MS

Minimum Number of Unique Peptide Sequences : 1

Minimum Number of Spectra : 1

Results: 9985 Genes

⊞ **Give this search a weight**

Step 5

STEP 5 : Api Specific

Included Species	pfal
Excluded Species	MAMM
Organism	Toxoplasma gondii GT1, Toxoplasma gondii ME49, Toxoplasma gondii VEG

Results: 2626 Genes

⊞ **Give this search a weight**

Plasmodb search

Step 1

STEP 1 : Exp Late Schiz

Experiment	Intraerythrocytic cycle transcriptome (3D7) sense strand
Direction	up-regulated
Reference Samples	17-25
Operation Applied to Reference Samples	minimum
Comparison Samples	40-5, 32-40
Operation Applied to Comparison Samples	maximum
fold difference >=	1.5
Protein Coding Only:	protein coding
Global min / max in selected time points	Don't care

Results: 2696 Genes

⊞ **Give this search a weight**

Step 2

STEP 2 : Pf Gam/Spo fc

Experiment	Sexual Cycle Expression
Direction	up-regulated
Reference Samples	Gametocyte
Operation Applied to Reference Samples	minimum
Comparison Samples	Sporozoite
Operation Applied to Comparison Samples	maximum
fold difference >=	2
Protein Coding Only:	protein coding

Results: 547 Genes

⊞ **Give this search a weight**

Step 3

STEP 1 : Signal Pep

Organism	Plasmodium falciparum, Plasmodium falciparum 3D7, Plasmodium falciparum IT
Minimum SignalP-NN Conclusion Score	3
Minimum SignalP-NN D-Score	0.5
Minimum SignalP-HMM Signal Probability	0.5
Matches any or all advanced parameters	any

Results: 2125 Genes

⊞ **Give this search a weight**

STEP 2 : Transmb Dom

Organism	Plasmodium falciparum
Minimum Number of Transmembrane Domains	1
Maximum Number of Transmembrane Domains	7

Results: 3106 Genes

⊞ **Give this search a weight**

Step 4

STEP 4 : Mass Spec

Experiment/Samples : **Blood stage phospho- and total proteome (3D7) (Boothroyd)**, schizont phosphopeptide-depleted, schizont phosphopeptide-enriched, Schizont cytosolic extract, **Infected erythrocytes from patient blood sample (Acharya et al.)**, P. falciparum infected erythrocytes from patient blood sample, **Intraerythrocytic stages phosphoproteome (3D7) (Pease et al.)**, intraerythrocytic stages, **Merozoite Proteome (3D7) (Kahn)**, merozoite proteome (3D7), **Merozoite phosphoproteome (3D7) (Lasonder et al.)**, Enriched merozoite phospho-proteins, **Parasite rupture from erythrocyte (D10) (Bowyer et al.)**, schizont, 42 h post-infection, schizont, 48 h post-infection, **Schizont Phosphoproteome (3D7)(2011) (Solyakov et al.)**, Enriched schizont phospho-proteins (2011), **Schizont Phosphoproteome (3D7)(2012) (Lasonder et al.)**, Enriched schizont phospho-proteins (2012), merozoite (synchronized), sporozoite, 14 day purified, trophozoite (synchronized), **Sporozoite Maturation Proteome (NF54) (Lasonder et al.)**, oocyst-derived sporozoite 13-14 days post-infection, oocysts 7-8 days post-infection, salivary gland sporozoites 18-22 days post-infection

Minimum Number of Unique Peptide Sequences : 2

Minimum Number of Spectra : 2

Results: 2572 Genes

⊞ **Give this search a weight**

Step 5

STEP 5 : Api Specific

Included Species	tgon
Excluded Species	MAMM
Organism	Plasmodium falciparum, Plasmodium falciparum 3D7, Plasmodium falciparum IT

Results: 1692 Genes

⊞ **Give this search a weight**
